# High Thermal Diffusivity in Thermally Treated Filamentous Virus-Based Assemblies with a Smectic Liquid Crystalline Orientation

**DOI:** 10.3390/v10110608

**Published:** 2018-11-02

**Authors:** Toshiki Sawada, Yuta Murata, Hironori Marubayashi, Shuichi Nojima, Junko Morikawa, Takeshi Serizawa

**Affiliations:** 1 Department of Chemical Science and Engineering, School of Materials and Chemical Technology, Tokyo Institute of Technology, Tokyo 152-8550, Japan; ymurata@polymer.titech.ac.jp (Y.M.); marubayashi@polymer.titech.ac.jp (H.M.); snojima@polymer.titech.ac.jp (S.N.); 2 Precursory Research for Embryonic Science and Technology, Japan Science and Technology, Saitama 332-0012, Japan; 3 Department of Materials Science and Engineering, School of Materials and Chemical Technology, Tokyo Institute of Technology, Tokyo 152-8550, Japan; morikawa.j.aa@m.titech.ac.jp

**Keywords:** filamentous virus, self-assembly, liquid crystal, thermophysical property, structural stability

## Abstract

Polymers are generally considered thermal insulators because the amorphous arrangement of the polymeric chains reduces the mean free path of heat-conducting phonons. Recent studies reveal that individual chains of polymers with oriented structures could have high thermal conductivity, because such stretched polymeric chains effectively conduct phonons through polymeric covalent bonds. Previously, we have found that the liquid crystalline assembly composed of one of the filamentous viruses, M13 bacteriophages (M13 phages), shows high thermal diffusivity even though the assembly is based on non-covalent bonds. Despite such potential applicability of biopolymeric assemblies as thermal conductive materials, stability against heating has rarely been investigated. Herein, we demonstrate the maintenance of high thermal diffusivity in smectic liquid crystalline-oriented M13 phage-based assemblies after high temperature (150 °C) treatment. The liquid crystalline orientation of the M13 phage assemblies plays an important role in the stability against heating processes. Our results provide insight into the future use of biomolecular assemblies for reliable thermal conductive materials.

## 1. Introduction

The increasing interest in flexible electronics has facilitated the development of new organic polymers that provide seamless integrated structure-property relationships required for a variety of efficient configurations in electronic devices, such as organic solar cells and organic light emitting diodes. Generally, the thermal conductivity of bulk organic polymers is very low due to strong phonon scattering caused by various defects and interfaces [1]. Typical methods for improving polymer thermal conductivity have often focused on the construction of composite materials, which contain additives (so-called fillers) such as metals or ceramics [2]. However, large amounts of fillers are required to sufficiently increase the thermal conductivity, which can significantly increase the materials cost and may change the original characteristics of the polymer, such as electrical, optical, and physical properties [3,4]. In contrast, the alignment of the polymer chains can greatly enhance their thermal conductivities along the axis of the polymer chains without the use of other additives [5,6]. Not only synthetic polymers [4,6,7] but also biomacromolecular proteins [8] can have high thermal conductivities, due to efficient phonon propagation along the oriented polymeric covalent bonds. Nevertheless, the practical applications of thermal conductive polymeric materials are still limited due to the inherent difficulties in processing the materials [9].

M13 bacteriophage (M13 phage), a filamentous virus, has a regularly assembled structure composed of multiple proteins and genomic DNA. In the past two decades, M13 phage has been used as various material components such as sensors, electronics, and devices [10,11,12,13,14,15,16,17,18]. M13 phage is an attractive biomaterial because of the ease by which its surface can be functionalized via genetic engineering [19] and/or chemical methods [20]. Furthermore, due to their uniform size, uniformity of dimensions, high aspect ratio (4.5 nm width and 900 nm length), dipole properties, and charge densities, M13 phage showed liquid crystalline properties with various phases in concentrated solutions [21,22,23,24]. Such liquid crystalline properties have been utilized for the construction of M13 phage-based materials with ordered structures [11,14,25,26,27,28,29,30,31,32,33]. Recently, we developed M13 phage-based assemblies with ordered structures in various scale (so called hierarchical assemblies) with high thermal diffusivity, even though the assemblies were based on non-covalent bonds, and found that macroscopic phage orientation in the assemblies is essential for high thermal diffusivity [34]. Despite the promising potential of biomacromolecular assemblies as thermal conductive materials, the stability against heating of the assemblies, which are generally regarded as low heat resistance objects, have rarely been investigated.

Biomacromolecular proteins are well known to denature upon heating, and typical denaturing temperature in an aqueous phase is approximately 60 °C, except for hyperthermophile proteins, viral assembled capsids, and similar systems [35,36]. To utilize M13 phage-based assemblies for reliable thermal conductive materials, the characterization of their thermal stabilities is an important requirement. Here we demonstrate the high structural stability against heating of M13 phage-based assemblies with high thermal diffusivities. Microscopic observations, infrared (IR) spectroscopic measurements, and small angle X-ray scattering (SAXS) measurements revealed that the molecular packing, secondary structures, molecular assembled structures, and macroscopic orientation of the assemblies had a smectic liquid crystalline orientation with little change upon thermal treatment at 150 °C. Importantly, the high thermal diffusivity of the assemblies was maintained after the thermal treatment. The thermal diffusivities of assemblies with nematic liquid crystalline orientation or with non-orientation are significantly decreased by thermal treatment; therefore, smectic liquid crystalline orientation plays an essential role in providing stability against heating. These results provide insight into the future use of biomolecular assemblies for reliable thermal conductive polymeric materials.

## 2. Materials and Methods

### 2.1. Preparation of M13 Phage-Based Films

The M13 phages were expressed using the Ph.D. Peptide Display Cloning System (New England Biolabs, Inc., Ipswich, MA, USA). Phagemid DNA was heat-shocked into competent *Escherichia coli* ER2738 cells. The M13 phages expressed were amplified using the *Escherichia coli* and purified by precipitation and re-dispersion procedures using a mixed solution composed of 5 *w*/*v*% poly(ethylene glycol) (average molecular weight of 7000–10,000) and 2.5 M NaCl. M13 Phage solutions (15 mg/mL, 500 µL) were mounted on glass plates circularly patterned with a highly water-shedding coating based on fluororesin (glass diameter: 15 mm, Matsunami Glass Ind., Ltd., Osaka, Japan) followed by incubation for 24 h at 25 °C in a dry atmosphere [34].

### 2.2. Polorized Optical Microscopy (POM) Observation

The phage films were set onto the stage of a polarized optical microscope (Eclipse LV100ND, Nikon). Then, the samples were observed at ambient temperature. Images with a sample rotation of 45° were recorded to identify the liquid crystalline-oriented structures.

### 2.3. Atomic Force Microscopy (AFM) Observation

The phage films were set onto the stage of an AFM. Then, the samples were observed. The AFM images were obtained using an SPM-9600 (Shimadzu, Kyoto, Japan) in tapping mode with standard silicon cantilevers. All images were scanned at a scan rate of at least 1 Hz with a maximum number of pixels (512 × 512).

### 2.4. Thermal Diffusivity Measurements

Thermal diffusivity (regarded as the ratio of the thermal conductivity of the material to the specific heat capacity) of the films in the thickness direction was measured using an ai-Phase mobile 1u (ai-Phase Co. Ltd., Tokyo, Japan) based on the temperature wave analysis method [37]. Thermal diffusivity (α) was calculated from the relationship of the square root of the angular frequency (√ω) and the phase delay (∆θ) of the temperature wave as shown in the following Equation (1):

(1) $$\Delta \theta =\;-\sqrt{\frac{\omega }{2\alpha }}d-\frac{\pi }{4}$$

where d is the thickness of the film.

### 2.5. SAXS Measurements

SAXS measurements of the phage films were performed at BL-10C (λ = 0.1488 nm) and 6A (λ = 0.1500 nm) of Photon Factory in KEK (Japan). The two-dimensional SAXS intensity was detected using PILATUS3 2M and 1M. A series of X-ray structure analyses were performed using homemade GUI software [38]. Full width at half maximum (FWHM) was determined by fitting with the pseudo-Voigt function to calculate the degree of orientation, which indicates the orientation states of the phage molecule in entire film, as in the following equation:

(2) $$\mathrm{Degree}\;\mathrm{of}\;\mathrm{orientation}=\frac{180-\mathrm{FWHM}}{180}$$

## 3. Results and Discussion

**Figure 1:**
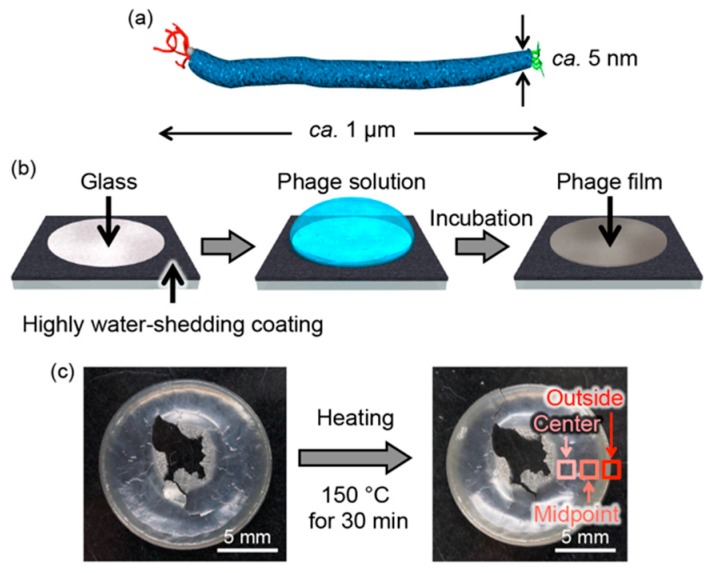
Schematic illustration of (**a**) M13 bacteriophage (M13 phage) and (**b**) preparation of the phage film. (**c**) Optical photographs of the as prepared (left) and the thermally treated (right) films composed of liquid crystalline oriented M13 phages.

M13 phage-based films of highly oriented liquid crystalline assembled structures with high thermal diffusivity were prepared according to a previously reported flow-induced method on a glass plate that had been circularly patterned with a hydrophobic coating based on fluororesin [34] (Figure 1b). We have previously found that the film thickness at the outside edge of the circular pattern (outside) is thicker than at the center (center) or part-way between the center and the outside (midpoint). This observation can be explained due to solute condensation that occurred by capillary flow induced by the differential evaporation rates across the drop at the outside of the pattern (so-called coffee-ring effect), resulting in different assembled structures [39]. It is noted that near the center, the film breaks when it is peeled off the glass substrate in order to measure the thermal diffusivity. In a control experiment, a non-oriented film composed of M13 phages was also prepared by a simple solution casting method. In military electronics, the desired high temperature target is 125 °C, whereas in automotive applications, it is 140 °C [40]. Therefore, the films were thermally treated at 150 °C for 30 min to evaluate their thermal stability. Rare differences were visually observed before and after thermal treatment (Figure 1c).

**Figure 2:**
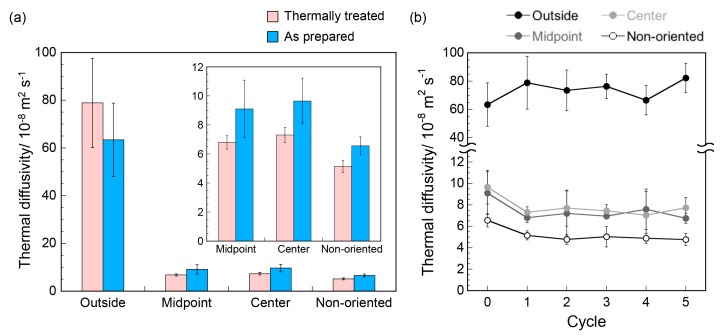
(**a**) Comparison of thermal diffusivity values of the as prepared and thermally treated M13 phage films. Thermal diffusivity values of the as prepared films were taken from ref. 34. Pink and blue bars represent thermal diffusivity values of the thermally treated and the as-prepared M13 phage films. Inset represents expanded view for the midpoint, center, and non-oriented M13 phage films. (**b**) Cyclic testing of thermal diffusivity of the films against thermal treatment. Thermal treatments and thermal diffusivity measurements were repeated for 5 times.

To characterize the effects of the thermal treatment on the thermal conductive properties of the M13 phage-based assemblies, thermal diffusivity values of the phage films at the three positions (outside, midpoint, and center) were measured by temperature wave analysis (Figure 2a). It was previously found that the thermal diffusivity value on the outside of the as-prepared film was approximately 10 times greater than that of non-oriented films due to the formation of extremely oriented structures in the assemblies, which might lead to a decrease in phonon scattering at structural defects [34]. The thermal diffusivity value at the outside of the thermally treated films was the same within experimental error as the as prepared films (that is, untreated films). On the other hand, the thermal treatment caused thermal diffusivity values to slightly decrease at the midpoint and center of the oriented films as well as across the non-oriented film (Figure 2a, inset). Importantly, thermal diffusivity values showed no further changes when cyclic testing of thermal treatments was performed, demonstrating thermal stability of the films (Figure 2b). Therefore, it was demonstrated that the assembled structures at the outside of the film are essential to maintain the thermal diffusivity after the thermal treatment (details are discussed later).

**Figure 3:**
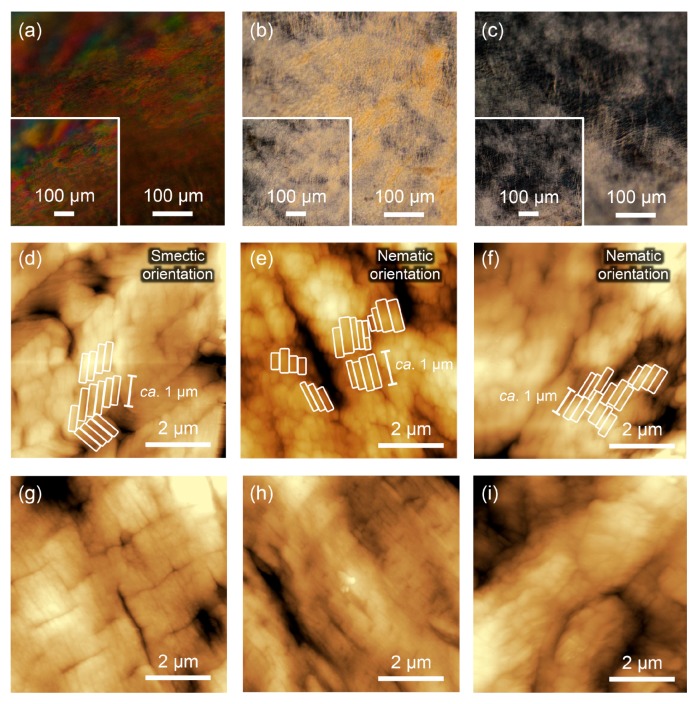
Microscopic observations of the M13 phage films prepared by flow-induced orientation methods. (**a**–**c**) Polarized optical microscopy (POM) images of the thermally treated M13 phage films. (**a**) Outside, (**b**) midpoint, and (**c**) center positions of the film were observed. Insets represent the POM images of the as prepared M13 phage films at the same positions. (**d**–**i**) Atomic force microscopy (AFM) images of the as prepared (**d**–**f**) and thermally treated (**g**–**i**) M13 phage films. Images (**d**,**g**) were taken from the outside, (**e**,**h**) the midpoint, and (**f**,**i**) the center of the film. Some of the observed domains have been highlighted by white oblongs in images (**d**–**f**). Scale bars are shown in the images.

POM was performed to evaluate the ordered structures of the M13 phages in the films before and after thermal treatment. In the POM images before thermal treatment, it was observed that the M13 phage films possessed differently oriented structures at the different positions on the film, as previously reported [34] (Figure 3a–c, inset). In brief, M13 phages on the film outside showed highly oriented smectic liquid crystal structures through a clearly layered birefringence (Figure 3a, inset). On the other hand, oriented structures consisting of nematic liquid crystals were observed at the midpoint and center positions of the film due to the absence of layered structures (Figure 3b,c, inset). The orientation regularity was higher at the outside of the film. Importantly, the POM images after thermal treatment exhibited the same oriented states at all three positions (Figure 3). Therefore, thermal treatment under the conditions applied here, (150 °C for 30 min) did not affect the liquid crystalline oriented states of M13 phages across the whole of the film at the POM scale.

The assembled structures of the M13 phages in the films were characterized by atomic force microscopy (AFM). Before thermal treatment, layered domains with a width of approximately 1 µm, which corresponds to the length of the M13 phage, were observed at the outside of the film, supporting smectic liquid crystal orientation on the micrometer scale, as previously reported [34] (Figure 3d). Similarly, nematic crystal orientation of the assembled domains with a width of 0.5–1 µm and a length of approximately 1 µm was observed at the midpoint and center positions (Figure 3e,f). Therefore, the M13 phages in the film were oriented in a plane direction and the orientation regularity was higher at the outside of the film, as observed in the POM studies. After thermal treatment, the layered domains at the outside were maintained even though the bending of the domains disappeared (Figure 3g), suggesting that the M13 phages still formed smectic liquid crystalline orientation. In contrast, the nematically-oriented domains at the midpoint and center positions were more packed or aggregated after the thermal treatment (Figure 3h,i), indicating that relaxation of the smectic-oriented domain structures was suppressed. These observations suggested that smectic liquid crystalline oriented states of the assembled M13 phages on the film outside appears to affect the thermal diffusivity values.

**Figure 4:**
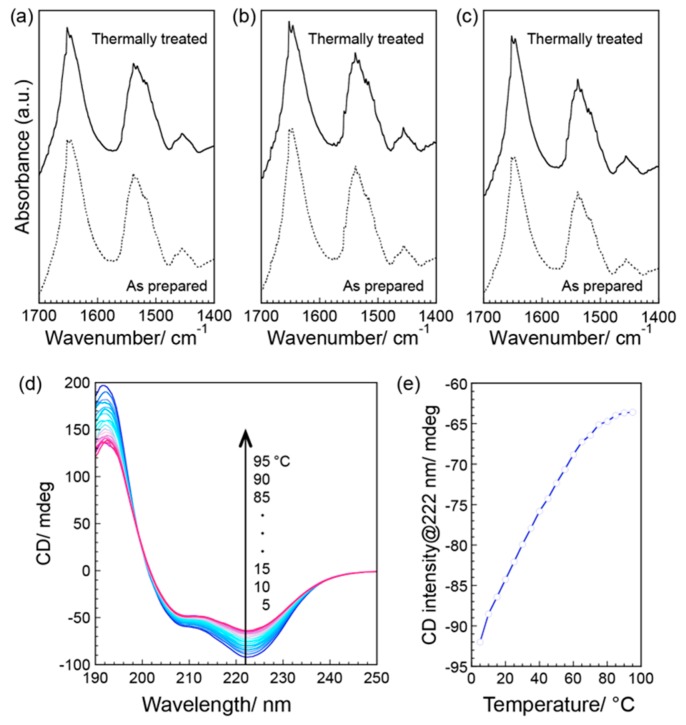
Secondary structural analyses of M13 phages. (**a**–**c**) Attenuated total reflection/infrared (ATR/IR) spectra of the (**a**) outside, (**b**) midpoint, and (**c**) center of the films. Solid and dotted lines represent the thermally treated and as prepared films, respectively. (**d**,**e**) Circular dichroism (CD) spectra of M13 phages in an aqueous solution. (**d**) CD spectra of M13 phages at different temperature (5–95 °C, every 5 °C). (**e**) CD intensity at 222 nm as a function of temperature. Phage concentration is 5 nM.

The secondary structures of the M13 phages in the film before and after the thermal treatment were characterized by attenuated total refraction (ATR)/IR spectroscopy measurements (Figure 4a–c). The IR spectra revealed two sharp peaks at approximately 1535 and 1630 cm^−1^ in all cases, which were primarily assigned to the α-helix structures of N-H bending (amide II) and C=O stretching (amide I) [41], respectively. After thermal treatment, peaks assignable to other secondary structures, such as the β-sheet conformation or random coil structures, were not observed. Importantly, the positions and half-band widths of the peaks were unchanged by the thermal treatment, suggesting that the original α-helical conformation was maintained. These results indicated that the slight structural changes, observed on the micrometer scale after thermal treatment, were not caused by changes in secondary structures. Such structural stability in proteins against high temperatures (150 °C) generally cannot be explained by the structural and/or conformational stability of proteins. On the other hand, the thermal stability of the secondary structures of M13 phage molecules in solution was characterized by circular dichroism (CD) spectroscopy (Figure 4d,e). The CD spectra revealed negative Cotton effects at 208 and 222 nm (Figure 4d), which were assigned to α-helical structures corresponding to the major coat proteins of M13 phages. The negative Cotton effects decreased with increasing temperature, demonstrating deformation of the α-helical structures. It is well known that the CD signal at 222 nm correlates to α-helical formation; thus, the signal values, as a function of the temperature, were obtained (Figure 4e). The decrease in the Cotton effect is saturated at approximately 80 °C, demonstrating that deformation of the M13 phage structures (i.e., denaturing) in a solution progressed sufficiently by heating at 80 °C. Therefore, the formation of liquid crystalline ordered structures in a solid state yielded structural stability against heating and resulted in the maintenance of high thermal diffusivity after thermal treatment.

**Figure 5:**
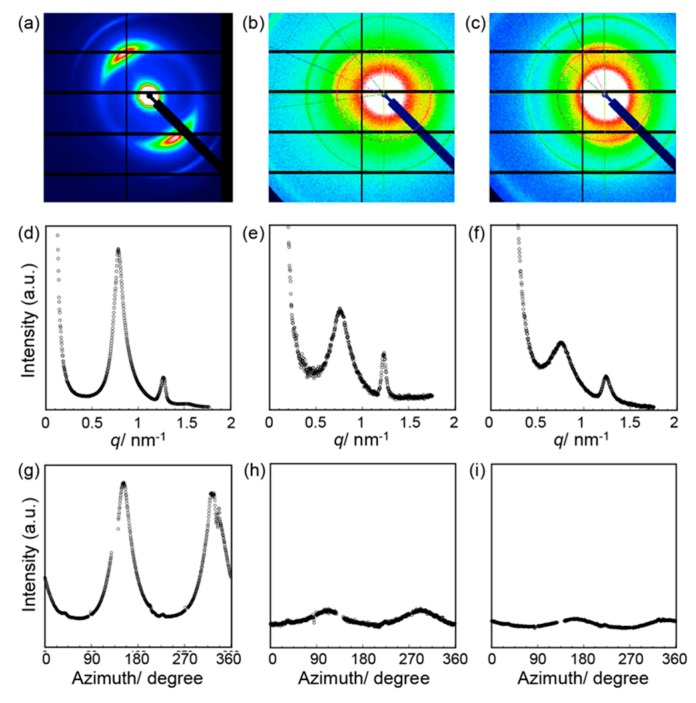
Small angle X-ray scattering analyses of the thermally treated M13 phage films. Two-dimensional (2D) patterns for the (**a**) outside, (**b**) midpoint, and (**c**) center of the film. The scattering curves for the (**d**) outside, (**e**) midpoint, and (**f**) center of the film. Azimuth scan of the primary peaks for the (**g**) outside, (**h**) midpoint, and (**i**) center of the film. Azimuth is zero in the upper direction from the center and increases in a clockwise direction in the 2D small angle X-ray scattering (SAXS) images.

SAXS experiments were performed to examine the stabilities based on a detailed structural characterization of the films (Figure 5a–c). In the resulting scattering profiles of the outside of the film, intense peaks at 8.21, 4.94, and 4.18 nm were observed with a reciprocal *d*-spacing ratio of 1:√3:2 (Figure 5d), which indicates hexagonally packed structures of the M13 phages. The scattering profiles for the midpoint and center of the film were also characteristic of hexagonally packed structures (Figure 5e,f). This packing structure of M13 phages at all positions on the thermally treated films was the same to that of the as prepared films, demonstrating that the packing of the M13 phages on a molecular level was essentially the same during the thermal treatment.

The degree of orientation, which represents the macroscopic orientation states, was calculated to quantitatively investigate the macroscopic orientation of the structures by full width at half maximum of the azimuth scan of the primary peaks at approximately 8 nm (Figure 5g–i), fitting with the pseudo-Voigt function according to a previous report [34]. The degree of orientation of the M13 phages in the entire film ranged from 0 to 1 (a higher value represents a higher orientation state). The resultant degree of orientation at the outside of the film after thermal treatment (0.76) was comparable to that of the outside of the as prepared films (0.78). These structural characterizations of the assemblies demonstrated that the smectic liquid crystalline oriented assemblies of M13 phages at the outside of the film were not affected by thermal treatment, indicating structural stability. In contrast, the degree of orientation values at the midpoint (0.18) and center (0.06) after thermal treatment were comparable or lower than those before treatment (0.19 and 0.01), respectively. The observed structural properties in the nematically oriented assemblies led to the scattering of phonons, possibly due to the presence of structural defects. Therefore, the hierarchical assembly of smectic liquid crystalline oriented M13 phages plays an important role in providing stability against heating in order to achieve reliable thermally conductive soft materials of biomolecular assemblies.

## 4. Conclusions

We investigated the thermal diffusivity of thermally treated M13 phage-based assemblies (films). Thermal diffusivity measurements demonstrated that the high thermal diffusivity of smectic liquid crystalline oriented assemblies of M13 phages (edge of the film) could be prepared through suitable convective assembly, remained unchanged by thermal treatment (150 °C for 30 min) and were approximately 10 times greater than those of nematic liquid crystalline oriented assemblies (midpoint and center of the film) and a non-oriented cast film. Thermal diffusivity values at the midpoint and center positions, as well as for the non-oriented film, slightly decreased upon thermal treatment. Structural characterization by microscopic observation, IR spectroscopy, and SAXS indicated that the structures of the smectic liquid crystalline assemblies with high thermal diffusivity were unaffected by the thermal treatment, which was in contrast to the nematic liquid crystalline oriented assemblies (with lower thermal diffusivity), in which the packing or aggregated states of the assembled domains changed over the micrometer scale. Even though the M13 phage is composed of biomolecular proteins, the smectic liquid crystalline orientation caused remarkable thermal stability. Additionally, our results indicating that ordered biomolecular assemblies were related to their thermal stability might help explain the evolution of life in high temperature environments, such as thermal vents. This biomolecule-based structurally regular assembly will open up attractive opportunities for the next generation of reliable thermal conductive materials composed of biomacromolecular assemblies.
